# Relevance of the Warburg Effect in Tuberculosis for Host-Directed Therapy

**DOI:** 10.3389/fcimb.2020.576596

**Published:** 2020-09-18

**Authors:** Bridgette M. Cumming, Hayden T. Pacl, Adrie J. C. Steyn

**Affiliations:** ^1^Africa Health Research Institute, Durban, South Africa; ^2^Department of Microbiology, University of Alabama, Birmingham, AL, United States; ^3^Centers for Free Radical Biology (CFRB) and AIDS Research (CFAR), University of Alabama, Birmingham, AL, United States

**Keywords:** tuberculosis, Warburg effect, macrophage, immunometabolism, host-directed therapy

## Abstract

Tuberculosis (TB) was responsible for more deaths in 2019 than any other infectious agent. This epidemic is exacerbated by the ongoing development of multi-drug resistance and HIV co-infection. Recent studies have therefore focused on identifying host-directed therapies (HDTs) that can be used in combination with anti-mycobacterial drugs to shorten the duration of TB treatment and improve TB outcomes. In searching for effective HDTs for TB, studies have looked toward immunometabolism, the study of the role of metabolism in host immunity and, in particular, the Warburg effect. Across a variety of experimental paradigms ranging from *in vitro* systems to the clinic, studies on the role of the Warburg effect in TB have produced seemingly conflicting results and contradictory conclusions. To reconcile this literature, we take a historical approach to revisit the definition of the Warburg effect, re-examine the foundational papers on the Warburg effect in the cancer field and explore its application to immunometabolism. With a firm context established, we assess the literature investigating metabolism and immunometabolism in TB for sufficient evidence to support the role of the Warburg effect in TB immunity. The effects of the differences between animal models, species of origin of the macrophages, duration of infection and *Mycobacterium tuberculosis* strains used for these studies are highlighted. In addition, the shortcomings of using 2-deoxyglucose as an inhibitor of glycolysis are discussed. We conclude by proposing experimental criteria that are essential for future studies on the Warburg effect in TB to assist with the research for HDTs to combat TB.

## Introduction

There is an unmet need for novel therapeutics against tuberculosis (TB), one of the leading killers among infectious diseases worldwide (WHO, 2019). While countries around the world set out to reduce TB mortality by 90% by 2030, a major breakthrough in TB therapy or immunization is needed by 2025 to meet this and other ambitious goals (WHO, 2019). Given that new antimicrobials effective against *Mycobacterium tuberculosis* (*Mtb*) are extremely limited, there is great interest in developing host-directed therapies (HDT) to tailor the responses of human cells to treat TB (Wallis and Hafner, 2015; Zumla et al., 2015). While HDT encompasses a broad range of targets from organ systems to subsets of cells, discussions of HDT in TB are generally focused on immune cells and their metabolism. In particular, inhibiting or potentiating aerobic glycolysis, also known as the Warburg effect, in immune cells have both been described as a potential approach to control *Mtb* infection, limit tissue damage, or to potentiate existing TB therapies (Shi et al., 2016; Krishnamoorthy et al., 2020). At present, the existing literature on the Warburg effect and its role in TB are in conflict, and therefore, the best approach for developing glycolysis-based HDTs for TB appears unclear. However, over the course of this review, we return to the origins of the Warburg effect, immunometabolism, and their application to TB to clarify apparent conflicts regarding the role of the Warburg effect in TB immunity. We posit that glycolysis in immune cells is essential for host protection in TB, and HDTs for TB should be aimed at enhancing glycolysis in the early stages of disease. Lastly, we will review several recent reports that *Mtb* inhibits glycolysis in infected host cells, highlighting a potential virulence strategy that has only recently been described for this ancient pathogen.

## Background: The Warburg Effect

In the early 1920's, Otto Warburg noticed that what set tumor tissue apart from normal tissue is that tumor tissue took up glucose and converted it to lactate in the presence of sufficient oxygen to convert glucose to CO_2_ (Warburg and Negelein, 1927), a phenomenon later termed the “Warburg effect” (Racker, 1972). Three metabolic features are essential for the definition of the Warburg effect, namely, (1) an increase in glucose uptake, (2) an increase in production of lactate, and (3) the availability of oxygen. Oxygen availability is often overlooked in this definition, which is important for distinguishing aerobic glycolysis from the increased glycolysis in response to hypoxia.

Warburg concluded that this aberrant metabolism demonstrated that “the respiration of all cancer cells is damaged” (Warburg, 1956). However, Warburg's own experiments using manometry on a variety of normal, embryonic and cancerous tissues, revealed equivalent, persistent oxygen consumption, and therefore equivalent respiration, in both tumor tissue and normal tissue (Warburg and Negelein, 1924; Koppenol et al., 2011; DeBerardinis and Chandel, 2020). Furthermore, studies from the past decade have indicated the importance of mitochondrial respiration and metabolism in tumor growth. For example, mitochondrial glutamine metabolism and transcription factors in addition to generation of mitochondrial reactive oxygen species are necessary for Kras tumorgenicity (Weinberg et al., 2010). In addition, tumor cells devoid of mitochondrial DNA acquire functional mitochondrial DNA from adjacent stromal cells *in vivo* to restore tumor growth (Tan et al., 2015). Intra-operative U-^13^C-glucose-tracing in human brain and lung tumors also demonstrate heterogenous labeling in tumors isolated from the same patient, including both glycolytic and tricarboxylic acid (TCA) cycle intermediates, providing evidence of a wide range of metabolic phenotypes in the tumors (Fan et al., 2009; Maher et al., 2012; Hensley et al., 2016). It is worth noting, however, that over the past decade many studies have documented oncogenic nuclear and mitochondrial DNA mutations in proteins involved in respiration, suggesting that Warburg's theory of “damaged respiration” applies to some cancers. In particular, mutations in genes coding for succinate dehydrogenase (Hao et al., 2009; Bayley and Devilee, 2010), fumarate hydratase (King et al., 2006), and isocitrate dehydrogenase (Parsons et al., 2008), in addition to heteroplasmy in mitochondrial DNA (He et al., 2010), have all been associated with tumorigenicity.

**Figure 1:**
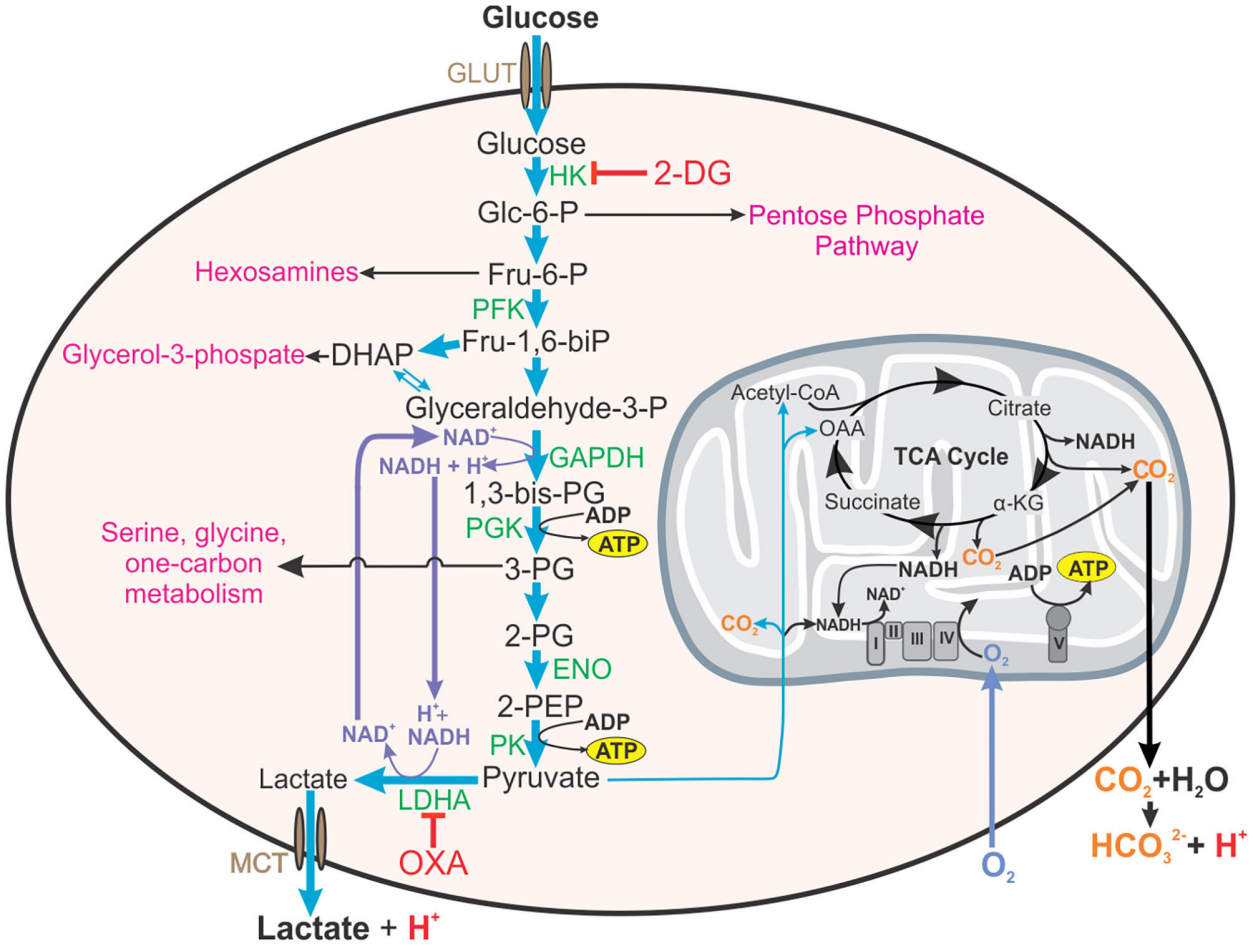
Roles of aerobic glycolysis. Aerobic glycolysis generates metabolic intermediates that are used in other biosynthetic pathways such as the pentose phosphate pathway, synthesis of hexosamines, glycerol-3-phosphate for fatty acid synthesis, amino acid synthesis, and one carbon metabolism. Aerobic glycolysis is also involved in maintaining redox balance (NAD^+^/NADH) in the cell. Inhibition of hexokinase (HK) in the first step of glycolysis by 2-DG will additionally inhibit these pathways and redox homeostasis. Thus, inhibitors reducing the glycolytic flux by acting on enzymes near the end of glycolytic pathway, such as OXA, on LDH, or LDHA knock-out mice will enable a more accurate assessment of the contribution of glycolysis to effector functions of the cell. This figure also demonstrates how both lactate from glycolysis and carbon dioxide generated from mitochondrial metabolism contribute to the acidification of the extracellular environment (ECAR). HK, hexokinase; 2-DG, 2-deoxyglucose; Glc-6-P, glucose-6-phosphate; Fru-6-P, fructose-6-phosphate; PFK, phosphofructokinase, Fru-1,6-biP, fructose-1, 6-bisphosphate; DHAP, dihydroxyacetone phosphate; GAPDH, glyceraldehyde phosphate dehydrogenase, 1,3-bis-PG, 1,3-bisphosphoglycerate; 3-PG, 2-PG, 3- or 2-phosphoglyerate; ENO, enolase; 2-PEP, 2-phosphoenolpyruvate; LDHA, lactate dehydrogenase A; OXA, sodium oxamate; OAA, oxaloacetate; α-KG, α-ketoglutarate.

Another proposed reason for the increased glycolytic rate in the presence of oxygen is the relationship that has been observed between glycolysis and rapidly dividing cells. Rapid glucose uptake and subsequent glycolysis enables cells to have high levels of glycolytic intermediates that feed into several pathways of macromolecular synthesis required for cellular division. For example, glucose-6-phosphate is the substrate for the pentose phosphate pathway that provides reducing equivalents (i.e., NADPH) and ribose for RNA and DNA synthesis; fructose-6-phosphate is used in the hexosamine pathway for protein glycosylation; 3-phosophoglycerate is a substrate for amino acid synthesis and one-carbon metabolism; and glycerol-3-phosphate is essential for the formation of phospholipids (Figure 1) (Hume and Weidemann, 1979; Newsholme et al., 1985; Vander Heiden et al., 2009). Interestingly, other studies have indicated that only a small percentage of the rate of glycolysis is utilized for biosynthetic purposes, with the rest of the carbon in glucose being converted and secreted as lactate (Hume et al., 1978). Conversely, high rates of glutamine utilization for anabolic carbon and nitrogen have also been reported in rapidly dividing cells (Hume and Weidemann, 1979; Newsholme et al., 1985; DeBerardinis and Cheng, 2010). Supporting this, Epstein et al. (2014) investigated the separation of energy demand and production in several cell lines representing the spectrum from normal breast epithelium to aggressive metastatic cancer. They found that under normoxic conditions, glycolysis supplies the rapid energy demands primarily to support membrane transport activities, which are enhanced for cell division, growth and migration, whereas slow-responding mitochondrial oxidative phosphorylation supplies chronic energy demand primarily for macromolecule synthesis (Epstein et al., 2014).

However, ultimately a tumor is more than just a three-dimensional collection of proliferating cells, they are dynamic complex structures where even individual cells have different metabolic requirements and functions. Recently, a study reported zonation of multiple metabolic niches in transplanted or natural glioblastoma tumors based on their relative distance from blood vessels (Kumar et al., 2019). The authors intravenously infused a fluorescent dye into the mice 1 or 5 min prior to harvesting the tumors and used flow-activated cell sorting and fractionation based on the uptake of the fluorescent dye to assess the relative distance from the blood vessels. From a spatial viewpoint, different areas of a tumor represent different degrees of perfusion, oxygen tension, nutrient supply, immune cell infiltration, and clonal evolution (Danhier et al., 2017). This results in metabolic adaptations such as a metabolic symbiosis where co-operation exists between cancer cells with different metabolic needs (Semenza, 2008; Sonveaux et al., 2008; Guillaumond et al., 2013), and adaptations to perfusion and acidosis (Sounni et al., 2014; Corbet et al., 2016). Therefore, the Warburg effect will likely only be functional in those cancerous cells that are actively dividing in the presence of oxygen. Likewise, the TB granuloma also has a three-dimensional structure and throughout the course of its evolution will alter degrees of perfusion, oxygen tension, nutrient supply, and infiltration of immune cells. These factors will also play a role in the metabolism of the immune cells constituting the granuloma.

## Background: Immunometabolism

A decade before Otto Warburg's seminal paper, a foundation was being laid for a new field of study investigating the intersection between metabolism and function in immune cells, which is today known as immunometabolism. These studies observed the consumption of oxygen (Grafe, 1911), the production of lactate from glucose (Levene and Meyer, 1912), and a high metabolic capacity in leukocytes (Maclean and Weir, 1915). Then, three years after the Warburg effect was described, Bakker published the first description of aerobic glycolysis in exudative leukocytes (Bakker, 1927). The following two decades saw a spirited debate over a variety of factors that influence leukocyte metabolism, including maturity (Kempner, 1939), injury (Bossa, 1937), and lineage (Soffer and Wintrobe, 1932). Much of the confusion over seemingly conflicting results likely stemmed from experimental methods and the species of origin for the cells under study (Beck and Valentine, 1953). Regardless, major tenants of the field of immunometabolism emerged during this period: exudative (i.e., inflammatory) leukocytes exhibit robust aerobic glycolysis (Bakker, 1927); myeloid cells, likely neutrophils, are more glycolytic than lymphocytes, likely naïve T cells (Soffer and Wintrobe, 1932); and aerobic glycolysis may be essential for host protection from bacterial infections (Kempner, 1939).

Following advances in experimental techniques, the latter half of the century saw these observations explored and expanded: protective host responses to bacteria were associated with increased aerobic glycolysis in neutrophils (Alonso and Nungester, 1956); key neutrophil effector functions, i.e., the oxidative burst and phagocytosis, are dependent on glycolysis (Sbarra and Karnovsky, 1959); and, under aerobic conditions, different phagocytes rely either on oxidative phosphorylation (OXPHOS) or glycolysis to carry out phagocytosis (Oren et al., 1963). This framework established metabolism as part of the effector process of neutrophils first, and other immune cells to follow (Oren et al., 1963; Wang et al., 1976; Hume et al., 1978). Such strides have been steadily made toward understanding the Warburg effect in immune responses, and since the turn of the millennium, we have begun to understand the upstream signaling pathways that regulate the induction of aerobic glycolysis in immune cells. This includes the discovery of toll-like receptor 4-mediated induction of aerobic glycolysis in lipopolysaccharide treated macrophages (Krawczyk et al., 2010; Rodriguez-Prados et al., 2010), as well as the identification of mTOR-HIF-1α (mammalian target of rapamycin; hypoxia inducible factor 1α) signaling pathway that mediates the metabolic transition to aerobic glycolysis in monocytes (Cheng et al., 2014).

Thus, the position of aerobic glycolysis in the stimulus-response arc of leukocytes has been slowly revealed over time. While we are always seeking an improved understanding of the Warburg effect, the field of immunometabolism has entered a new phase: application. Across scientific disciplines, investigators are framing scientific questions about pathogenesis and treatment of disease within our understanding of the contribution of aerobic glycolysis to immunity. Researchers are excited by the possibility of new targets for therapeutic intervention, or a greater understanding of pathogenesis in diseases with excessive or insufficient inflammation, including numerous cancers (Xie et al., 2014; Seth et al., 2017), autoimmune conditions (Rhoads et al., 2017), and infections (Kaufmann et al., 2018). The idea of modulating aerobic glycolysis within host cells to treat infections represents a major development in the approach to infectious diseases and one that is welcomed, given the emergence of drug-resistant bacteria. Not surprisingly, the Warburg effect has become a topic of great interest in the study of TB.

## The Warburg Effect in TB: Lesions and Lungs

The earliest studies of the Warburg effect in TB focus predominantly on lesion-level metabolism, or in other words, the sum of the metabolic activity of the immune cells gathering at the site of TB pathology. The techniques used to assess the metabolism of TB lesions included transcriptomics or metabolic profiling of lungs from TB mouse and rabbit models, and granulomatous tissue isolated from the TB guinea pig model and human TB patients. Interestingly, increased glucose uptake, the first component in the definition for the Warburg effect, was first appreciated in TB lesions by positron-emission tomography (PET) imaging (Kim et al., 2008; Hahm et al., 2010). PET imaging is an important complement to computed tomography (CT) and traditional X-ray imaging for the diagnosis of TB and is also a potential approach to monitoring patient responses to TB therapy (Davis et al., 2009). It works by infusing a patient with a radioactive glucose analog, 2-deoxy-2-[fluorine-18] fluoro-D-glucose (^18^F-FDG), which then circulates around the body and accumulates in regions with a high demand for glucose. While it has not been directly measured, it is likely that the regions of tissue with access to ^18^F-FDG also have some access to oxygen, as these regions must have access to systemic circulation to take up 18F-FDG. Then it is reasonable to propose that the metabolic activity observed via PET imaging could represent the Warburg effect. In the context of TB, ^18^F-FDG uptake serves as proxy for inflammation, potentially linking glucose metabolism to inflammation at the site of TB pathology. Though this may be the case in the short term, increased glucose uptake in TB lesions is also associated with the recovery of *Mtb* mRNA, suggestive of ongoing bacterial replication (Malherbe et al., 2016).

It is also worth mentioning that there is great heterogeneity among lesions in terms of their spatial distribution, cellular composition, overall structure, and pathophysiology within the microenvironment of the human tuberculous lung (Lenaerts et al., 2015; Hunter, 2016, 2018; Hunter et al., 2018; Pacl et al., 2018; Reddy et al., 2018; Rahman et al., 2020; Wells et al., 2020). While PET imaging can certainly capture some TB lesions, it may miss others, including hypoxic lesions. Factors such as granuloma pathophysiology and structure, spatial distribution of lesion in relation to the vasculature and airways, the nutritional environment and metabolism of constituent cells, or even simply the perfusion of diseased lung may play a role in the sensitivity of PET scanning in detecting TB pathology. Preoperative PET imaging followed by detailed assessment of the gross, or histopathology in lung resections from TB patients may be a powerful approach to understanding the *in vivo* metabolism across the full spectrum of TB pathology observed only in humans.

Although the TB mouse model has proved to be useful in TB drug development (Davies et al., 2007), the most commonly used laboratory mouse strains, C57BL/6 and BALB/c, do not develop granulomas with central necrosis and fibrosis that are characteristic of human TB (Orme, 2003; Bharath and Balasubramanian, 2011; Hunter, 2016, 2018; Hunter et al., 2018). This alone suggests that metabolism observed in these mouse TB lungs would differ to that in human TB. However, the first study proposing the Warburg effect was induced in TB, was conducted by Shi et al. examining the bioenergetic metabolism of C57BL/6 mouse lungs after a low-dose of aerosol *Mtb* H37Rv (Shi et al., 2015). RNA-sequencing of the infected mouse lung tissue at 12, 18 and 30 days revealed an upregulation of genes encoding glycolytic enzymes (Figure 1): hexokinases (*Hk2* and *3*), phosphofructokinase (*Pfk*) family 1 and 2, glyceraldehye-3-phosphate dehydrogenase (*Gapdh*), phosphoglycerate kinase (*Pgk1*), enolase (*Eno1*), and lactate dehydrogenase A (*Ldha*); glucose transporters (*Glut1, 3* and *6*); a transporter for lactate (*Mct4*); an H^+^-ATPase involved in cytosolic pH homeostasis; and Hif-1α (regulatory unit of HIF-1, a transcription factor). HIF-1α is known to regulate energy metabolism and cellular adaptions under hypoxia, but it is also involved in regulating inflammatory processes under normoxia (Imtiyaz and Simon, 2010). However, the expression levels of some of these glycolytic genes were only upregulated by ~2 fold, such as *Glut1, Glut3*, the PFK-1 encoding genes, *Gapdh, Pgk1, Eno1*, and *Ldha*, with only *Hk3* demonstrating the most significant 15-fold increase in transcript levels (Shi et al., 2015). Furthermore, the authors observed a concurrent downregulation of genes encoding enzymes involved in the TCA cycle for pyruvate oxidation and OXPHOS in the mitochondria. Importantly, immunofluorescence microscopy revealed increased protein production of HK3, LDHA, the H^+^-ATPase (ATP6a3), and HIF-1α in the mouse lung macrophages and CD3^+^ T cells (Shi et al., 2015). Using these findings, the authors concluded that infection with *Mtb* induces the Warburg effect in the mouse lungs.

Unlike the C57BL/6 and BALB/c mouse models, the rabbit model of TB simulates some features of human TB, including the formation of well-differentiated granulomas that range from necrotic, caseous, cavitating to healing lesions (Subbian et al., 2011). Analysis of the lung transcriptome of rabbits aerobically infected with the hypervirulent strain of *Mtb*, HN878, shows upregulation of gene networks involved in the inflammatory response between 4 and 8 weeks of infection (Subbian et al., 2011). Similar to mice, an upregulation of “Warburg effect-associated gene expression,” including some, but not all glucose transporters, glycolytic enzymes, monocarboxylate transporters, HIF-1α, and its regulators, was observed (Shi et al., 2016). When the disease in the rabbit progressed with a sustained high bacillary load, intensified disease pathology and a compromised host response at 12 weeks (Subbian et al., 2011), the authors report that the gene expression of “Warburg effect” enzymes and HIF-1α still remained at elevated levels (Shi et al., 2016).

Progressing to human pulmonary TB, RNA transcriptomics of cavitary lung granulomas of human patients with active TB compared to uninvolved portions of the same lungs (Subbian et al., 2015) also demonstrated increased gene expression of glucose transporters, glycolytic enzymes, monocarboxylate transporters, H^+^-ATPase, HIF-1α, and its regulators (Shi et al., 2016). Likewise, the authors concluded the Warburg effect is present in human granulomas of patients with active TB. However, an upregulation in the expression of the monocarboxylate transporter, MCT1, was also observed. As MCT1 has a high affinity for lactate, the authors propose that increased lactate metabolism may also take place in the human granulomas (Subbian et al., 2015). This suggests metabolism different to the Warburg effect in more advanced stages of TB disease that may be due to the depletion of glucose and contribute to the development of cavitary granulomas. This could bring into question the presence of the Warburg effect in the human TB cavitary granulomas (Shi et al., 2016).

In line with this, potential metabolic evidence of increased glycolysis in TB was provided by metabolic profiling of infected mouse lungs using NMR. Increased lactate and decreased glucose and glycogen were detected in the lungs, liver, spleen and serum of C57BL/6 mice 4 weeks after infection with 200 CFU of *Mtb* H37Rv via the aerosol route (Shin et al., 2011). In contrast, metabolomics of lungs collected at 4- and 9-weeks post infection from C57BL/6 mice infected with a high dose (~2500 CFU) of *Mtb* H37Rv via the intratracheal route revealed only a moderate increase in lactate with disease progression (Fernandez-Garcia et al., 2020). These differences may be due to a different route of infection with a much higher dose of *Mtb*, in addition to the fact that three mass spectrometry platforms, namely, capillary-electrophoresis-time-of-flight, gas-chromatography-quadrupole-time-of-flight, and liquid chromatography-quadrupole-time-of-flight, were used to analyze the metabolites. Furthermore, analysis of the metabolomics at 4- and 9-weeks post infection revealed progressive catabolism with carbohydrate depletion, fluctuations in energy-storage lipids and a continuing increase in amino acids and oligopeptides with disease progression. This suggests that the high dose of *Mtb* infection induces wasting with TB progression (Fernandez-Garcia et al., 2020) and that the Warburg effect might be absent at advanced stages of disease in mice.

When organized granulomas that developed in Hartley guinea pigs infected with *Mtb* H37Rv in visibly identified granulomatous tissue were profiled using NMR at 15, 30, and 60 days relative to uninfected lung tissue, a progressive increase in lactate levels was detected (Somashekar et al., 2011). However, increased lactate levels alone in lesion-level analysis are not adequate to infer the presence of the “Warburg effect” as granulomas induced by *Mtb* infection of guinea pigs, rabbits, and non-human primates have been shown to be hypoxic (Via et al., 2008). Thus, in this case, the increased lactate levels are likely indicative of glycolysis under hypoxia, and not the Warburg effect. Strikingly, granulomas in both H37Rv and *Mtb* Erdman infected wild type C57BL/6 mice appear not to be hypoxic (Aly et al., 2006; Tsai et al., 2006), hence supporting the Warburg effect described in *Mtb*-infected C57BL/6 mouse lungs. Thus, lactate measurements in conjunction with measurements of glucose uptake and knowledge (or measurement) of the oxygen tension in the environment are preferable to demonstrate the Warburg effect (Table 1). Importantly, in the case of studies at the lesion-level or organ level, it is important to note that these analyses include both the extracellular and intracellular concentrations of the metabolites in a collection of many cell types.

**Table 1:** Experimental guidelines to investigate the Warburg effect in TB.

**Points to consider**	**Description/notes**
**Model system**	**Macrophages:** Bioenergetic responses of murine and human macrophages are different (Michelucci et al., 2013; Vijayan et al., 2019) Differentiation of monocytes into macrophages must be described in detail. Macrophages with different ontogenies differ in their bioenergetic metabolism (Huang et al., 2018) ***In vivo*** **(mouse) studies** Transgenic knockout mice, whole body versus cell/organ-specific; can measure metabolism in its totality, and gain further knowledge of mechanism in the wild-type (Hackett et al., 2020; Rahman et al., 2020)
**Uptake of glucose**	• Measure glucose concentration in supernatant before and after an infection period or at different time intervals of infection to determine the rate of glucose consumption • Vary glucose concentrations as the glucose concentration in commercially available media exceeds what is present in the human body • Measure glucose concentration in the organs of animal models
**Metabolism of glucose**	• Using ^13^C_6_-glucose, examine isotopolog distribution to trace the flux of glucose through central metabolites and amino acids, and calculate the total abundance of the metabolites and amino acids. • Use ^13^C_1, 2_-glucose and ^13^C_6_-glucose to calculate fraction of glucose carbons flowing through the PPP versus glycolysis (Lee et al., 1998; Bruntz et al., 2017) • Examine absolute and relative levels of glycolytic, PPP and TCA metabolites of two conditions examined (Rahman et al., 2020)
**Lactate secretion**	• Measure secreted and intracellular lactate concentrations. • Determine ^13^C_6_-enrichment of intracellular/extracellular lactate of cells cultured with ^13^C_6_-glucose. • Determine pyruvate/lactate ratio. • Measure glycoPER and not ECAR by extracellular flux analysis (Cumming et al., 2018)
**Availability of oxygen**	***In vivo:*** • Proximity of cells/lesions from blood vessels/capillaries (Kumar et al., 2019) • Measuring pO_2_ (Aly et al., 2006; Via et al., 2008)***In vitro:*** • The pO_2_ levels in cell cultures in conventional CO_2_ incubators is considered to be dependent on cell type and the cell's preference for OXPHOS, cell density, depth of media, media composition and time in culture (Zeitouni et al., 2015; Place et al., 2017; Ast and Mootha, 2019)
**Infection**	• Infection with a virulent, live strain of *Mtb*. • Killed *Mtb*, γ-irradiated *Mtb* or stimulation with lysates should not be used as a substitute for live virulent *Mtb*.
***In vivo*** **granulomas and/or lesions**.	• The pathophysiology of animal model and human lesions differ significantly (Tsai et al., 2006; Hunter, 2016, 2018; Hunter et al., 2018; Hunter and Actor, 2019) • The pros and cons of metabolic pathways generated from -omic technologies (e.g., transcriptomics, proteomics, epigenetics, and metabolomics) should be carefully considered. Generally, in eukaryotes and bacteria there is a relatively poor correlation between transcripts and corresponding protein levels (Pearson correlation coefficient of ~0.4, Vogel and Marcotte, 2012).**Immunohistochemistry (IHC) of metabolic markers:** • Quantification of the IHC signal is difficult as it reflects antibody sensitivity and specificity; relative differences can only be measured on the same section/slide. • IHC is not necessarily an indication of protein abundance or enzymatic activity.**Histopathology:** • Using IHC (or other related techniques), it is recommended that metabolic markers be examined within the context of the histopathological spectrum of TB including, but not limited to: • Necrotic granulomatous inflammation: ▪ Cavitational, Caseative necrosis, Coagulative necrosis, Fibrinoid necrosis • Non-necrotic granulomatous inflammation: ▪ Granulation tissue, Granulomatous inflammation, Fibrous tissue/lamellar fibrosis
**Controls**	• Control cells or other *in vitro* model systems infected with killed *Mtb* is preferred to using uninfected controls. • Non-activated neutrophils and macrophages (depending on their lineage) have short life spans and for this reason, we propose comparing host cells infected with virulent live *Mtb*, with host cells infected with dead *Mtb:* ▪ Including *M. bovis* BCG in infection experiments could be beneficial. ▪ In case of genetically modified knockout mice, infected knockout mice should be compared with infected wild-type mice and not with uninfected mice.

In summary, the above transcriptomic studies only demonstrate the intent of the cell in the TB lung or granulomas, but do not provide definitive evidence of metabolism as insinuated by a correlation of 0.4 between mRNA abundances and protein concentrations (Vogel and Marcotte, 2012). Thus, transcriptomics alone, are insufficient to conclude that the “Warburg effect” is present and concurrent metabolic analysis of these samples would have provided more compelling evidence of the final metabolic products and the extent of glycolysis. In addition, addressing or measuring the oxygen tension in these lesions or granulomas would have conclusively validated the “Warburg effect” as the Warburg effect *is* dependent on the status of the oxygen tension (Table 1). Increased glycolysis under hypoxia is the natural metabolic response of most cells. However, increased glycolysis *in the presence of oxygen* is what renders the Warburg effect as a unique metabolic exception that can be modulated by host-directed therapies. For this reason, compelling evidence indicating that the Warburg effect controls *Mtb* infection is essential to identify effective HDTs for TB.

## The Warburg Effect in TB: Macrophage Functions

Lesion-level assessments have since been enhanced by the study of the single cells that make up the granuloma. In particular, the role of aerobic glycolysis in the metabolism and effector functions of macrophages responding to infection with *Mtb* became the focus of the TB field. A crucial advance in our understanding of the role of aerobic glycolysis in TB immunity was that aerobic glycolysis is required to mediate the protective effects of interferon gamma (IFN-γ) in TB (Braverman et al., 2016). The authors identified the transcription factor, HIF-1α, as a mediator of IFN-γ-induced gene expression, and that IFN-γ-treated mouse bone-marrow derived macrophages (BMDMs) lacking HIF-1α had a higher bacillary burden than HIF-1α-sufficient BMDMs. Metabolic profiling of *Mtb*-infected HIF1α^−/−^ BMDMs treated with IFN-γ revealed reduced glucose uptake and lactate production, suggesting attenuated aerobic glycolysis. Causally linking the two, treatment of BMDMs with the glucose uptake inhibitor 2-deoxyglucose (2-DG) abrogated the reduction in bacillary burden conferred by IFN-γ. This mechanistic analysis was supported by *in vivo* studies showing that whole-body HIF-1α^−/−^ mice are more susceptible to TB (Braverman et al., 2016), and this was later enhanced by two studies showing that myeloid-specific conditional HIF-1α knockout mice are also more susceptible to TB (Osada-Oka et al., 2019; Resende et al., 2020). One of these studies also indicated that the transcription of lactate dehydrogenase A was controlled by HIF-1α in *Mtb* infected BMDMs independent of INF-γ, and demonstrate that HIF-1α reduces the pyruvate levels in the macrophages, which is used as a preferable source for intracellular *Mtb* replication over glucose (Osada-Oka et al., 2019). However, this study supports previous findings (Braverman et al., 2016) that the treatment of BMDMs with IFN-γ induces a greater increase in the secreted lactate levels and further reduces the bacillary loads than that observed in untreated BMDMs (Osada-Oka et al., 2019).

It is worth noting, however, that the *in vivo* component of these studies does not directly test the role of aerobic glycolysis in TB. HIF-1α regulates the expression of a wide range of genes that may be important in TB outcomes, including VEGF and erythropoietin in addition to glycolytic enzymes and LDHA (Forsythe et al., 1996; Semenza et al., 1996; Ebert and Bunn, 1999). In addition, the IFN-γ/HIF-1α axis has also been found to redistribute macrophage lipids into lipid droplets as part of the host-defense strategy (Knight et al., 2018), which is completely aside from aerobic glycolysis. Toward this end, Huang et al. investigated *Mtb* replication *in vivo* and found that *Mtb* preferentially replicates in alveolar macrophages, which have a metabolism characterized by OXPHOS, over the more glycolytic infiltrating macrophages (Huang et al., 2018). Upon treating *Mtb*-infected mice with 2-DG, the highly glycolytic subset of infiltrating macrophages was selectively depleted and the burden of *Mtb* increased. On the other hand, selective depletion of alveolar macrophages via intranasal delivery of clodronate-laden liposomes diminished *Mtb* burden. This suggests that glycolysis in the infiltrating interstitial macrophages in the mouse lung enables these cells to control *Mtb* growth, while fatty acid oxidation in the alveolar macrophages permits *Mtb* growth in these cells.

Defining this causal link is difficult in part because glycolysis is extremely challenging to study mechanistically. These foundational studies all use 2-DG to experimentally manipulate glycolysis. While 2-DG inhibits hexokinase in the first step of glycolysis–this in turn limits the glycolytic intermediates that are utilized in many anabolic pathways of the cell, such as the pentose phosphate pathway, resulting in off-target effects on metabolism. To understand the role of glycolytic activity in cell function, then, only flux through the pathway should be disturbed and not the availability of its constituent metabolites. One approach to do this is to target the highly conserved, indirect regulator of glycolytic flux, lactate dehydrogenase (LDH). LDH oxidizes NADH to NAD^+^ while reducing pyruvate to lactate, replenishing the NAD^+^ that is required for maximal flux through glycolysis (Figure 1). Therefore, targeting LDH activity, can selectively decrease carbon flux through glycolysis without decreasing the availability of glycolytic intermediates for downstream pathways. Inhibitors of LDH, such as sodium oxamate (Figure 1), can be used to selectively decelerate glycolysis (Lustig and Redman, 1980). Another approach taken by Osada-Oka et al. (2019), was to generate LDHA-deficient RAW264.7 cells that were impaired in their ability to control *Mtb* infection. While this is an immortalized, murine cell line that is relatively disconnected from humans, it provides an important validation that aerobic glycolysis is important in the anti-*Mtb* effector functions of macrophages.

## Macrophage Response to *Mtb* Infection

While reports suggested that *Mtb* may induce aerobic glycolysis in macrophages upon infection (Gleeson et al., 2016; Lachmandas et al., 2016; Vrieling et al., 2020), our work (Cumming et al., 2018) and that of others (Hackett et al., 2020) has recently demonstrated that *Mtb* inhibits aerobic glycolysis in infected host macrophages. These differences may be due to varying protocols used for the differentiation of monocytes into macrophages. But further experimentation in these studies supporting the *Mtb* induction of aerobic glycolysis, and new studies by the same groups, utilized an avirulent strain of *Mtb*, H37Ra, or γ-irradiated dead *Mtb* (Gleeson et al., 2016; Phelan et al., 2020) or *Mtb* lysate (Lachmandas et al., 2016; Vrieling et al., 2020) for the infection of the macrophages. Past studies have shown that in comparison to *Mtb* H37Rv, H37Ra has decreased survival in macrophages (Mackaness et al., 1954) and induces Th1 responses in contrast to Th2 responses generated by H37Rv (Freeman et al., 2006). Furthermore, distinct differences have been observed in the bioenergetic metabolism induced by infection with live virulent *Mtb* H37Rv, dead *Mtb* or non-virulent, vaccine strain, *M. bovis* Bacillus Calmette-Guérin (BCG) (Cumming et al., 2018). Thus, claiming findings deduced from macrophages infected with avirulent or dead *Mtb* strains as evidence of response to infection with live virulent *Mtb* should be interpreted with caution. Furthermore, in these bioenergetic studies (Gleeson et al., 2016; Lachmandas et al., 2016; Hackett et al., 2020; Phelan et al., 2020; Vrieling et al., 2020), extracellular acidification rate (ECAR) was used as a measure of glycolysis. Extracellular acidification is due to the protons produced by the secretion of lactate from glycolysis in addition to the protons produced from the reaction of CO_2_, which is generated by reactions in the TCA cycle, with water forming carbonic acid (Mookerjee et al., 2015) (Figure 1). Thus, ECAR alone is not an accurate measurement of glycolysis. For this reason, the glycolytic proton efflux rate (glycoPER), which is the difference between the total proton efflux rate and the mitochondrial proton efflux rate that is calculated from the measurements of both the oxygen consumption rate (OCR) and ECAR, was introduced (Mookerjee and Brand, 2015) and is therefore, a more accurate indicator of the rate of glycolysis. Using glycoPER as a measure of glycolysis, studies in human monocyte-derived macrophages (hMDM) indicated that infection with *Mtb* (H37Rv) decelerated glycolysis 24 h post infection. Conversely, infection with the vaccine strain, *M. bovis* Bacillus Calmette-Guérin (BCG) and dead *Mtb* (heat-killed) did indeed increase glycolysis in hMDMs (Cumming et al., 2018). Given the importance of aerobic glycolysis in macrophages, this may represent a strategy used by virulent *Mtb* to subvert the immune response to establish an infection.

Another recent study also observed restriction of glycolysis in mouse BMDM infected with viable *Mtb* relative to infection with γ-irradiated dead *Mtb* after 24 h (Hackett et al., 2020). Using BMDM from microRNA-21-deficient mice, the authors demonstrated that this reduction of glycolysis was due to upregulation of anti-inflammatory microRNA-21, which targets the muscle-phosphofructokinase isoform (PFK-M) at the first rate-limiting and committed step of glycolysis (Tanner et al., 2018). The reduced glycolysis induced by *Mtb* decreases the production of pro-inflammatory mediators, e.g., IL-1β, and facilitates the growth of mycobacteria in macrophages (Hackett et al., 2020).

Furthermore, in another study investigating the role of host H_2_S in TB, we provide further evidence for *Mtb* induced suppression of glycolysis in mouse peritoneal macrophages (Rahman et al., 2020). Peritoneal macrophages derived from cystathionine-γ-lyase (CSE^−/−^) deficient and wild-type C57BL/6 mice were infected with *Mtb* H37Rv for 24 h, followed by metabolite extraction. Analysis of the metabolites using LC-MS/MS revealed that the abundance of several glycolytic intermediates in CSE^−/−^ *Mtb* H37Rv infected peritoneal macrophages was 2-3-fold greater than that in infected wild-type macrophages. These findings demonstrated how increased host H_2_S production resulting from *Mtb* infection suppressed glycolysis in the infected macrophages (Rahman et al., 2020).

These conflicting results regarding the rate of glycolysis of *Mtb* infected macrophages could be explained by a recent review that the mouse macrophage has a biphasic metabolic response during the progression of infection with *Mtb* (Shi et al., 2019). Using differential gene analysis of transcriptome databases of mouse BMDMs, the authors suggest that an early phase (4–8 h post-infection) is characterized by a pro-inflammatory polarization accompanied by HIF-1α mediated aerobic glycolysis, upregulation of oxidative, and antioxidative defense responses and synthesis of bioactive lipids. This is followed by a later adaptation/resolution phase (24–48 h post-infection) in which there is a down regulation of immune response genes and a transition from glycolysis to mitochondrial oxidation in mouse BMDMs. Another study using a mouse model of progressive pulmonary tuberculosis, also demonstrated that the *Mtb* H37Rv infection is divided into two phases (Baay-Guzman et al., 2018). During the first phase, corresponding to the first month, granulomas are produced. During the second phase, extensive pneumonia is developed. Immunohistochemical staining demonstrated strong HIF-1α nuclear staining in activated macrophages located in granulomas during the first phase. During the late phase, higher HIF-1α expression was observed in foamy macrophages and some lymphocytes in the pneumonic areas. When a potential HDT, 2-methoxyestradiol that inhibits HIF-1α, was administered during the first phase, the lung bacillary loads were greater than the control group, and a greater area of the lung surface was affected by pneumonia. But when 2-methoxyestradiol was administered in the second phase, both the lung bacillary load and lung surface area affected by pneumonia were significantly reduced (Baay-Guzman et al., 2018). However, it is not known if this biphasic response is exhibited in infected human macrophages.

Much of the contradiction observed in literature is also due to the macrophage models that are used in the studies, such as primary human versus murine macrophages. A recent report indicated prominent metabolic differences between mouse BMDMs and hMDMs that were activated with lipopolysaccharide (LPS) (Vijayan et al., 2019). For instance, lactate production and ECAR were significantly increased with a considerable decrease in OCR in the LPS activated mouse BMDMs, whereas a slight decrease in ECAR with no changes in the lactate production and OCR was observed in the LPS-activated hMDMs. Furthermore, activated hMDMs depended only on OXPHOS for ATP production, whereas activated BMDMs depend on both glycolysis and OXPHOS for ATP production (Vijayan et al., 2019). Another distinguishing feature between mouse and human macrophages is that LPS activated murine macrophages produce high levels of itaconic acid. In mouse macrophages, itaconic acid has anti-mycobacterial properties (Michelucci et al., 2013) as well as anti-inflammatory effects induced by inhibiting succinate dehydrogenase, reducing succinate levels and IL-1β production (Lampropoulou et al., 2016); and preventing the formation of mitochondrial reactive oxygen species by reverse electron transport at complex I (Mills et al., 2016; Scialo et al., 2017). However, intracellular concentrations of itaconic acid in LPS- treated human macrophages were two orders of magnitude lower than that in mouse BMDMs (Michelucci et al., 2013), and one cannot conclude without evidence that these much lower levels of itaconic acid will induce similar effects in human macrophages. Thus, it is essential that findings from *Mtb* infected mouse macrophages should be verified in human macrophages.

## T Cell Response to *Mtb* Infection

Our knowledge of the role of adaptive immunity within the TB lesion is limited. T-cell mediated immunity is considered to be protective in TB as CD4^+^ T cells, specifically IFN-γ producing Th1 cells, are essential for the adaptive immune response against TB in both mice and humans (Cooper, 2009). Also, CD8^+^ T cells help to control *Mtb* infection by perforin-facilitated cytolysis of infected macrophages and direct killing of *Mtb* (Woodworth and Behar, 2006). However, even less is known about the modulation of the immunometabolism associated with these T cell responses in TB.

Immunometabolic studies with healthy T cells have shown that marked aerobic glycolysis, the Warburg effect, is associated with T cell activation by T cell-receptor ligation and binding with co-stimulatory molecules to induce an anabolic program to increase biomass for proliferation (MacIver et al., 2011). Furthermore, distinct metabolic programs differentiate T cells into their lineages and determine their effector functions, for example, Th1, Th2, and Th17 cells rely strongly on glycolysis (Michalek et al., 2011). However, the effector functions of the T cells can be limited by environmental chemical signals or checkpoint regulators on the cell surface, such as PD-1, CTLA-4, LAG-3, and 3B3 (Patsoukis et al., 2015). PD-1 alters metabolic programming during T cell effector differentiation by inhibiting glucose transport and glycolysis and inducing lipolysis and β-oxidation of endogenous fatty acids. In contrast, CTLA-4 inhibits glycolysis without enhancing fatty acid oxidation (Patsoukis et al., 2015).

In TB, the development of the granuloma can potentiate microenvironments that contribute to T cells' hypo-responsiveness as observed in T cells infiltrating the cancer tumor microenvironment (Chang et al., 2015; Siska and Rathmell, 2015). In chronic *Mtb* infection of mice, increased expression of inhibitory receptors, TIM3, PD-1, and LAG-3, was observed in functionally exhausted CD4^+^ and CD8^+^ T cells (Jayaraman et al., 2016). Another study from our group reported that significantly increased expression of the inhibitory receptors, PD-1 and CTLA-4 on *Mtb* specific CD8^+^ T cells after 12 weeks of H37Rv *Mtb* infection (100 CFU) of C57BL/6J mice coincided with decreased glucose uptake in these cells and reduced effector function and polyfunctionality (Russell et al., 2019). Moreover, extracellular flux analysis revealed that both the mitochondrial respiration (OCR) and ECAR of these CD8^+^ T cells deteriorated with disease progression to metabolic “quiescence” after 12 weeks of infection. This was supported by similar intracellular lactate levels in the CD8^+^ T cells isolated from uninfected and 12-week *Mtb* infected mice. Interestingly, an anti-diabetic drug, metformin, which has been shown to reduce bacillary burden in combination with anti-*Mtb* targeting drugs in *Mtb* infected mice (Singhal et al., 2014), restored both OCR and ECAR in mouse CD8^+^ T cells after 7 weeks of *Mtb* infection to that of uninfected mice (Russell et al., 2019). These findings provide further evidence of potential HDTs restoring the bioenergetic metabolism of immune cells in TB. Thus, we need to advance our knowledge of the adaptive immune response to TB as the T cell response in TB may provide a further target for the use of HDTs to improve the immune response to TB thereby shortening treatment duration.

## Conclusion

The Warburg effect in cells is characterized by the increased uptake of glucose and the increased production of lactate–in the presence of oxygen (DeBerardinis and Chandel, 2020). However, elevated glucose uptake and lactate production are also “hallmarks” of glycolysis under hypoxia, thus the presence of oxygen is the defining factor of the Warburg effect. Initial studies investigating the metabolic responses in TB using organs or lesions isolated from animal models of TB and TB patients pointed to a Warburg-like metabolism being prominent in *Mtb* infection. However, the presence or availability of oxygen was often overlooked in these studies. PET imaging demonstrates the uptake of glucose by certain lesions in humans and non-human primates and the presence of oxygen is accepted by virtue of the close proximity of the lesions to blood vessels to take up ^18^F-FDG for the PET imaging and thus oxygen. However, lactate levels in these TB lesions are unknown. The “Warburg effect” in TB was also concluded by *in vitro* studies in macrophages that reported increased ECAR from extracellular flux analysis of *Mtb* infected macrophages. However, extracellular acidification is generated from both glycolysis and respiration, and glycoPER is a more accurate measure of the proton production rate due to glycolysis alone. Furthermore, these findings are complicated by the use of an attenuated strain of *Mtb*, H37Ra, γ-irradiated dead *Mtb* or heat-killed dead *Mtb*, instead of live virulent *Mtb*, for the infection of the macrophages. The use of primary murine or human macrophages adds to the diverse range of findings in immunometabolic studies of *Mtb* infection. For these reasons, we propose experimental criteria that should be considered to demonstrate the Warburg effect in TB studies (Table 1).

Conversely, some studies in mice do suggest that aerobic glycolysis does play an important role in the control of *Mtb* in mice. Using HIF-1α knockout mice, it was demonstrated that HIF-1α, which upregulates the expression of glycolytic genes, is essential for the control of *Mtb* infection (Braverman et al., 2016). Another study reported that the ontology of the interstitial macrophages and hence their increased glycolytic activity, controlled *Mtb* growth early during infection (Huang et al., 2018). However, a recent review of transcriptome databases of *Mtb* infected BMDMs suggests that the Warburg effect may only be prominent at initial stages of infection (Shi et al., 2019). Interestingly, decelerated glycolysis was observed in *Mtb* infected hMDMs after 24 h of infection (Cumming et al., 2018), miRNA-21 has been reported to limit glycolysis in *Mtb* infected macrophages by repressing PFK-M expression (Hackett et al., 2020) and increased host hydrogen sulfide generation also depressed glycolysis in the *Mtb* infected macrophages (Rahman et al., 2020). In the case of the adaptive immune response to TB, treatment of *Mtb* infected mice with metformin, considered to activate GAPDH and AMPK, restored both the respiratory and glycolytic functions of the CD8^+^ T cells. This finding demonstrates proof of principle that pharmacological manipulation of immunometabolism using HDTs is a viable strategy to simplify and reduce TB treatment duration.

Thus, HDTs aimed at signaling pathways that promote aerobic glycolysis in the early stages of infection may stimulate improved proinflammatory and Th1 immune responses to *Mtb* infection. However, extended upregulated pro-inflammatory responses have been associated with immunopathogenicity, thus the timing and duration of these HDTs will be crucial (Baay-Guzman et al., 2018; Rao et al., 2019; Young et al., 2020). Timing and dosing of appropriate HDTs is actually of utmost importance as indicated by studies in cancer (Rothschilds and Wittrup, 2019) and inflammatory diseases (Baraliakos and Braun, 2018). Furthermore, the ontology and spatial location of the immune cells in the lung and granuloma and perfusion of the diseased tissue will also determine the efficacy of HDTs. Thus, for HDTs promoting aerobic glycolysis to be considered as potential adjunctive treatment of TB, deeper knowledge of the timing, spatial location, ontology and identity of immune cells involved in the Warburg effect to control *Mtb* infection will need to be more conclusively established.

## Author Contributions

All authors listed have made a substantial, direct and intellectual contribution to the work, and approved it for publication.

## Conflict of Interest

The authors declare that the research was conducted in the absence of any commercial or financial relationships that could be construed as a potential conflict of interest.
